# One Health Surveillance of Antimicrobial Resistance in the Eastern Mediterranean: The Blackchin Guitarfish as a Case Study

**DOI:** 10.1029/2025GH001680

**Published:** 2026-04-10

**Authors:** J. Lellouche, H. Di Castro, N. Maschiah, S. Paikin, M. Zvereva, D. Tchernov, A. Scheinin, E. Cohen, D. Meron

**Affiliations:** ^1^ Adelson School of Medicine Ariel University Ariel Israel; ^2^ Clinical Laboratories Department Sanz Medical Center Laniado Hospital Netanya Israel; ^3^ Koret School of Veterinary Medicine Hebrew University of Jerusalem Jerusalem Israel; ^4^ Kadas Nexus Leon H. Charney School of Marine Sciences University of Haifa Sdot Yam Israel

**Keywords:** antimicrobial resistance, one health, *Glaucostegus cemiculus* blackfish guitarfish

## Abstract

Antimicrobial resistance (AMR) poses a global One Health challenge, linking human, animal, and environmental health. Marine environments and organisms are increasingly recognized as reservoirs of antimicrobial‐resistant bacteria and mobile genetic elements. This study investigates the prevalence of antibiotic non‐susceptible bacteria and resistance genes in juvenile *Glaucostegus cemiculus* blackchin guitarfish along the Israeli Mediterranean coast. Between 2023 and 2024, 19 specimens were sampled from Ma'agan Michael, Acer, and Evtach. Swabs from skin, gills, and mouth were cultured on selective and chromogenic media, followed by identification using matrix‐assisted laser desorption/ionization—time‐of‐flight (MALDI‐TOF MS) and antimicrobial susceptibility testing. Resistance genes were screened by quantitative PCR (qPCR), with CTX‐M beta‐lactamases (*bla*_CTX‐M_) variants sequenced and phylogenetically analyzed. A total of 162 bacterial isolates were obtained, of which 54% were identified to 26 species across eight families, primarily Staphylococcaceae (39%) and Bacillaceae (36%). Several clinically relevant pathogens were detected, including *Staphylococcus aureus*, *Pseudomonas spp*., and *Escherichia coli*. Reduced susceptibilities were observed in 31 isolates from 10 specimens, with multidrug resistance identified in *P*. *mendocina*, *P*. *stutzeri*, and *E*. *coli*. Skin samples yielded the highest proportion of resistant isolates. Importantly, the *bla*_CTX‐M‐185_ extended‐spectrum β‐lactamase gene was detected in six individuals, with sequences closely related to those of human‐associated strains, suggesting anthropogenic origins. These findings demonstrate that juvenile guitarfish harbor clinically significant resistant bacteria and genes, highlighting the marine environment as a potential reservoir of AMR. Integrating endangered species into AMR surveillance highlights the importance of for environmental monitoring and conservation strategies within a One Health framework.

## Plain Language Summary

Our study applies a One Health perspective to investigate antimicrobial resistance in marine environments. Using the blackchin guitarfish (*G*. *cemiculus*) as a model, we sampled individuals along the Israeli Mediterranean coast and analyzed bacterial isolates from the gills, skin, and mouth. We detected multiple clinically relevant pathogens, including *Staphylococcus* spp., *Pseudomonas* spp., and *E*. *coli*, several of which exhibited resistance to key antibiotics such as β‐lactams, cephalosporins, and carbapenems. Notably, the *bla*_CTX‐M‐185_ gene was detected in multiple samples, highlighting the presence of mobile resistance elements and the potential for horizontal gene transfer. To our knowledge, this is the first study to evaluate AMR prevalence in *G*. *cemiculus* in the Eastern Mediterranean, providing novel insights into marine reservoirs of antimicrobial resistance. Our findings demonstrate that marine organisms can serve as reservoirs for AMR, linking human activity (e.g., wastewater discharge), wildlife health, and ecosystem stability. Furthermore, our findings provide valuable data relevant to global AMR surveillance, illustrating the interconnectedness of human, animal, and environmental health. This work underscores the importance of integrated environmental monitoring within a One Health framework for assessing public health risks and marine ecosystem integrity.

## Key Points

- Juvenile blackchin guitarfish along the Israeli Mediterranean coast harbor clinically relevant antibiotic‐resistant bacteria
- Multidrug‐resistant strains and the blaCTX‐M‐185 gene indicate potential anthropogenic sources of AMR in marine environments
- Monitoring bacteria and resistance genes in marine organisms indicates ecosystem health and supports a One Health approach

## 1 Introduction

### 1.1 The Role of Antimicrobial Resistance (AMR) in One Health

Antimicrobial resistance (AMR) represents a central One Health challenge at the interface of human, environmental, and wildlife health (CDC, 2024; Destoumieux‐Garzón et al., 2018). Excessive and poorly regulated antimicrobial use in human and agricultural systems promotes the emergence of resistant bacteria and resistance genes, which are increasingly disseminated into coastal and marine environments via wastewater discharge, agricultural runoff, and other anthropogenic pathways (Berendonk et al., 2015; Velazquez‐Meza et al., 2022). In low‐ and middle‐income countries, high antimicrobial use combined with weak regulatory frameworks promotes the environmental release of antibiotics and resistant bacteria, fostering persistent reservoirs of antimicrobial resistance beyond clinical settings (Ehsan, 2025; Ehsan, Ibrahimkhil, et al., 2025, Ehsan, Wardak, et al., 2025). Horizontal gene transfer enables antimicrobial resistance genes to persist and disseminate across environmental reservoirs, facilitating their long‐term maintenance beyond clinical settings (Endale et al., 2023; Hernando‐Amado et al., 2019).

### 1.2 AMR in the Marine Environment

Coastal and marine environments receive continuous inputs of antibiotics, resistant bacteria, and resistance genes through wastewater discharge, agricultural runoff, and riverine transport, facilitating the establishment of environmental reservoirs of antimicrobial resistance. While AMR has been documented in marine sediments and water columns, systematic surveillance through marine wildlife remains limited, particularly in mobile and long‐lived species that integrate exposure across space and time (Berendonk et al., 2015; Le Quesne et al., 2018). Antimicrobial resistance has been widely documented in marine sediments, water columns, and select marine organisms, with numerous studies reporting resistant bacteria in benthic substrates and filter‐feeding taxa such as bivalves (Al‐Sarawi et al., 2022; Albini et al., 2022; Habibi et al., 2025). Marine mammals have been used as sentinel species for antimicrobial resistance, reflecting cumulative exposure to contaminated coastal environments and highlighting the potential for wildlife‐mediated integration of environmental AMR signals (Gross et al., 2022; Norman et al., 2021). However, such efforts have focused largely on mammals, leaving other long‐lived and mobile marine taxa underrepresented in AMR surveillance frameworks. In the Eastern Mediterranean, high‐risk antimicrobial resistance has been documented in nearshore marine and riverine environments, including the widespread detection of carbapenemase‐producing Enterobacterales in coastal waters of Israel (Cohen et al., 2020). These findings highlight the marine environment as an important reservoir of clinically relevant resistance; however, the extent to which such resistance is incorporated into marine wildlife remains poorly understood.

### 1.3 *G*. *cemiculus* (Blackchin Guitarfish) as a Potential Sentinel Species for AMR Surveillance

The blackchin guitarfish (*G*. *cemiculus*) is a coastal, benthic elasmobranch whose life cycle is closely associated with shallow nearshore habitats subject to intense anthropogenic influence (Notarbartolo di Sciara et al., 2016; Serena, 2005). Adults are primarily benthic and confined to continental shelf habitats at depths of up to ∼160 m, with limited movement along coastlines (Compagno, 1999; Gong, 2022), whereas early life stages are restricted to extremely shallow nearshore nursery grounds within the surf zone, where exposure to coastal anthropogenic inputs is maximal (Azrieli et al., 2024). As a long‐lived and mobile species, *G*. *cemiculus* integrates environmental exposure across space and time, making it a suitable sentinel species for assessing antimicrobial resistance in coastal ecosystems. Despite growing evidence of environmental AMR in the Eastern Mediterranean, the presence and characteristics of resistant bacteria and resistance genes in elasmobranchs remain largely unexplored. In this study, we aimed to characterize the occurrence of antibiotic non‐susceptible bacteria and clinically relevant antimicrobial resistance genes in juvenile *G*. *cemiculus* from coastal habitats along the Israeli Mediterranean coast, within a One Health framework.

## 2 Materials and Methods

### 2.1 Sample Collection

**Figure 1:**
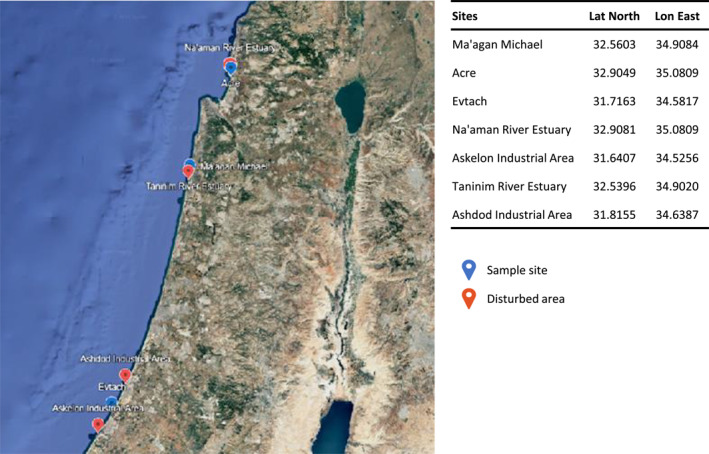
Map of the sampling sites (blue markers) and their respective adjacent with disturbed area (red markers).

Between 2023 and 2024, a total of 19 juvenile *G*. *cemiculus* were sampled as part of an exploratory study providing an initial characterization of antimicrobial resistance patterns in this species. Specimens were collected from three coastal sites along the Israeli Mediterranean shoreline: Ma'agan Michael on the central coast (*n* = 13), Acer on the northern coast (*n* = 1), and Evtach on the southern coast (*n* = 5) (Figure 1).

Sampling was conducted in accordance with ethical guidelines and relevant permits from the Nature and Parks Athorities and the fishery department (permit # 2024/43590). Net deployments were conducted at selected locations throughout the study area, following a consistent protocol. Nets were set 10–30 m from shore at depths up to 1.3 m (as described in Azrieli et al., 2024). Captured specimens were gently removed by hand and placed in a container with 140 L of seawater and sand.

**Figure 2:**
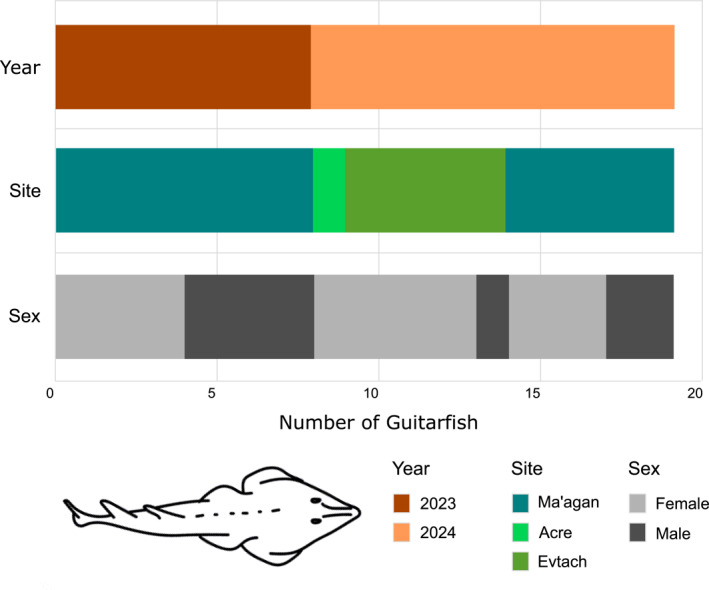
Distribution of sampled *G*. *cemiculus* by year, site, and sex (*n* = 19).

Each specimen was measured on a flat tray for total length (TL), disc length, and disc width to the nearest millimeter and weighted using a waterproof stainless steel weighing scale to the nearest gram. Sex was determined based on the presence or absence claspers. The sampled individuals had an average TL of 33.5 ± 3.9 cm and an average body mass of 164.4 ± 41.7 g. The sample comprised 12 females (63%) and seven males (37%). Eight individuals were sampled in 2023 and 11 in 2024. Detailed metadata for each specimen, including sampling site, year, sex, and morphometric measurements, are provided in Figure 2 and Table 1.

**Table 1:** Metadata for Juvenile *G*. *cemiculus* Specimens

N	Guitarfish_ID	Sampling date (year)	Sampling site	Sex	TL (cm)	Weight (gram)
1	G1A	2023	Ma'agan Michael	Female	34.4	N/A
2	G2	2023	Ma'agan Michael	Female	33.5	90
3	G5	2023	Ma'agan Michael	Female	34.1	100
4	G9	2023	Ma'agan Michael	Female	32.7	90
5	G3	2023	Ma'agan Michael	Male	32.6	100
6	G4	2023	Ma'agan Michael	Male	31.7	80
7	G6	2023	Ma'agan Michael	Male	32.7	90
8	G8	2023	Ma'agan Michael	Male	33.6	110
9	G1B	2024	Acer	Female	22.6	33
10	G18	2024	Evtach	Female	40.4	180
11	G20	2024	Evtach	Female	35	142
12	G21	2024	Evtach	Female	32.5	109
13	G22	2024	Evtach	Female	37.2	134
14	G19	2024	Evtach	Male	34	112
15	G40	2024	Ma'agan Michael	Female	33.4	101
16	G41	2024	Ma'agan Michael	Female	38.3	148
17	G43	2024	Ma'agan Michael	Female	35.6	129
18	G42	2024	Ma'agan Michael	Male	35.7	145
19	G44	2024	Ma'agan Michael	Male	26.4	54

*Note*. Year, sampling site, total length (cm), weight (g), and sex of the 19 specimens included in this study and sampled for the three anatomical sites: skin, gills, and mouth.

**Figure 3:**
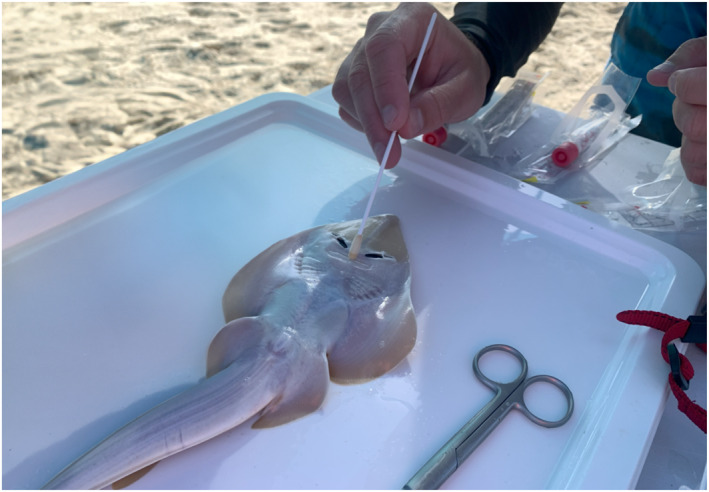
Representative mouth sampling using swab.

Samples were collected from three anatomical sites: skin, gills, and mouth (Figure 3). Sampling was performed using sterile swabs with liquid Amies transport media (Eswab®, Copan). Following collection, swabs were stored in a cooled container at approximately 4°C and transported to the laboratory for further processing.

### 2.2 Non‐Susceptible Isolates Identification and Characterization

Samples were enriched by transferring 1 mL of transport media to 50 mL of brain heart infusion‐broth medium (BHI, Hy Laboratories, Rehovot, Israel). BHI enrichment significantly improves the sensitivity of bacterial detection rates (Liss et al., 2013; Peretz et al., 2016). Broths were incubated aerobically for 18hr at 25 ± 2°C. Following enrichment, the samples were visually checked for turbidity, indicating bacterial growth, before being inoculated on chromogenic and selective agar plates. We evaluated the presence of bacteria harboring (a) extended‐spectrum beta‐lactamases (ESBL, CHROMagar™ ESBL, Hy Laboratories) and carbapenemases (CHROMagar™ mSuperCarba, Hy Laboratories) enzymes that confer resistance to most beta‐lactam antibiotics including carbapenems; (b) carbapenem resistance in Acinetobacter spp (CHROMagar™ MDR Acinetobacter, Hy Laboratories); (c) vancomycin resistance in Enterococcus spp (CHROMagar™ VRE, Hy Laboratories); (d) methicillin resistance in *Staphylococcus aureus* (CHROMagar™ MSSA/MRSA, Hy Laboratories) and (e) the presence of Candida spp (CHROMagar™ Candida Plus, Hy Laboratories). In addition, unselective media will be used to evaluate the bacterial burden and diversity (Marine broth agar, Blood TSA agar, MacConkey agar, Hy Laboratories). All the plates were incubated aerobically at 25°C for 18 hr. Suspected colonies were identified by morphology and color.

Identification to the species level was performed by Matrix‐Assisted Laser Desorption/Ionization Time‐of‐Flight Mass Spectrometry (MALDI‐TOF MS, BioMérieux). The spectra generated by VITEK™ MS PRIME was analyzed by the VITEK™ MS Software (version 1.1.2) and the VITEK™ MS IVD Database 3.3. Spectra that provide confidence values of 60%–99.9% for the similarity to a reference species in the database are considered high‐confidence identifications. Identifications are defined as low discrimination when a spectrum matches to two, three, or four species equally. No ID is reported when there is no match in the database or when more than four species are matched, which results in a confidence value  <60%. If no identification or a low confidence score was obtained, the pathogen was classified as a non‐human pathogen and removed from the study.

Bacteria including *Staphylococcus spp*., *Pseudomonas spp*., *E*. *coli*, and *P*. *agglomerans* were exposed to a targeted range of selected antibiotics to assess their resistance profiles. Minimum Inhibitory Concentrations (MICs) were determined biochemically using a VITEK™2 system (bioMérieux), and antimicrobial susceptibilities were interpreted according to Clinical and Laboratory Standards Institute M100 guidelines (Blosser, S. CLSI M100). For each isolate, susceptibility was categorized as susceptible (S), intermediate (I), or resistant (*R*) (Table S1). In the case of *Bacillaceae spp*., *Aeromonadaceae spp*., *Vibrionaceae spp*., or *Shewanellaceae spp*. Antimicrobial susceptibility testing was not performed due to the unavailability of appropriate commercial testing panels.

### 2.3 AMR‐Related Genes Detection

Broths were also evaluated by screening the presence of CTX‐M beta‐lactamases (*bla*_CTX‐M_), *bla*_IMP_, *bla*_KPC_, *bla*_NDM_, *VanA*, *VanB*, and *bla*_VIM_ genes using quantitative PCR (qPCR, Allplex Entero‐DR, Seegene, Korea). Gene variant for CTX‐M positive samples was determined by amplification using specific primers (CTX‐M‐F: ATGTGCAGYACCAGTAARGTKATGGC and CTX‐M‐R: TGGGTRAARTARGTSACCAGAAYCAGCGG, length 593 bp) (Sidjabat et al., 2009). PCR products were sequenced by Sanger (HyLabs, Rehovot, Israel). Presence of AMR among sex was evaluated using a Fisher's exact test and among individuals using a Mann–Whitney U test. For both tests, a *p* < 0.05 was considered significant.

### 2.4 Phylogenetic Analysis of CTX‐M Gene

To examine the evolutionary relatedness of the CTX‐M resistance genes, sequences were aligned with homologous gene sequences retrieved from the NCBI database. For comparison, the 10 most similar sequences available in NCBI (based on BLAST results), as well as additional sequences previously identified in marine environments, were included in the analysis. Phylogenetic reconstruction was conducted by MEGA‐X using the Maximum Likelihood method under the Tamura 3‐parameter model with a proportion of invariable sites (+I) (Kumar et al., 2018).

## 3 Results

A total of 19 G. *cemiculus* were included in the analysis, representing both sexes and a narrow size range typical of juvenile individuals (Table 1). Bacterial isolates were obtained from these individuals and subsequently identified and characterized, providing an overview of the microbial composition and enabling assessment of antimicrobial resistance patterns.

### 3.1 Bacterial Isolates Identification

**Figure 4:**
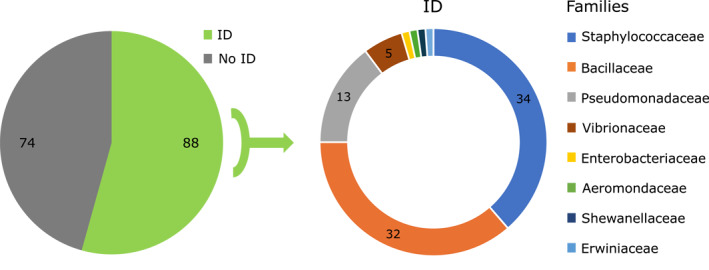
(a) Total number of bacterial isolates identified as human pathogens and unidentified from sampled *G*. *cemiculus* (b) Number of identified bacteria clustered by family level.

A total of 162 bacterial isolates were obtained from 19 G. *cemiculus*. Among 162 isolates, 88 (54%) were successfully identified to the species level, while 74 (46%) remained unidentified. The identified isolates were assigned to eight bacterial families and 26 Species, most of which have pathogenic potential for humans (Table S2). The most frequently identified families were Staphylococcaceae (*n* = 34, 39%) and Bacillaceae (*n* = 32, 36%), together accounting for over three‐quarters of all identified isolates. Other represented families included Pseudomonadaceae (*n* = 13, 15%), Vibrionaceae (*n* = 5, 6%), and single isolates from Aeromonadaceae, Erwiniaceae, and Shewanellaceae (each *n* = 1, 1%) (Figure 4, Table S3).

Among 162 isolates, 46 were isolated from gill samples, 55 from skin samples, and 61 from mouth samples. From isolates that were successfully identified (*n* = 88), 26 were isolated from gill samples, 27 from skin samples, and 35 from mouth samples. The mouth yielded the highest number of identified isolates (40%), followed by the skin (31%) and gills (29%).

**Figure 5:**
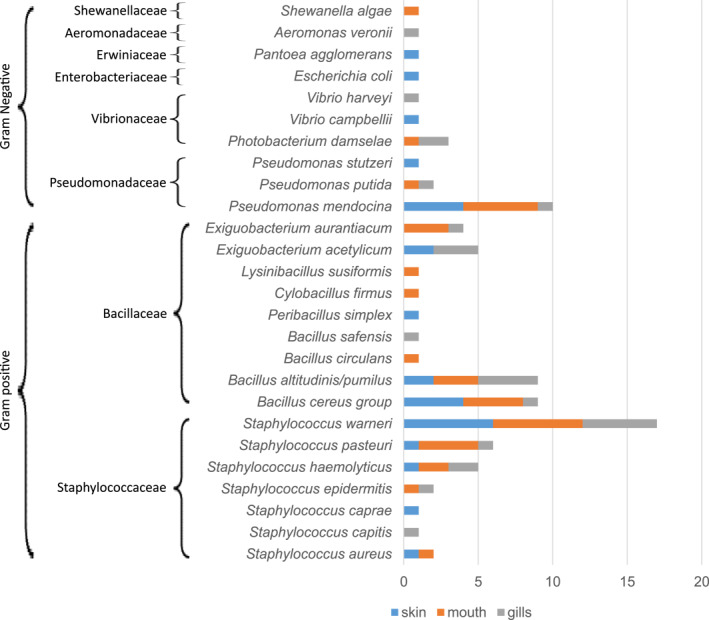
Bacterial isolates from sampled *G*. *cemiculus*, grouped by Gram‐staining (brackets on the left) and identified to species level. Horizontal bars represent the frequency of each taxon across three anatomical sites: skin (blue), mouth (orange), and gills (gray). Values on the *x*‐axis indicate the number of isolates recovered per taxon per anatomical site.

Among bacterial isolate families, 2 were Gram‐positive and 6 were Gram‐negative. The Gram‐positive group was consisted of nine isolates from the Bacillaceae family and seven from the Staphylococcaceae family. The Gram‐negative group included a more diverse set of taxa: 3 Pseudomonadaceae, 3 Vibrionaceae, and single representatives of Enterobacteriaceae, Shewanellaceae, Aeromonadaceae, and Erwiniaceae (Figure 5).

### 3.2 AMR Evaluation

Among *Staphylococcus spp*. Isolates (*n* = 33), the susceptibility testing revealed distinctive resistance patterns. All isolates of *S*. *aureus* (6%, 2/33), *S*. *capitis* (3%, 1/33), *S*. *caprae* (3%, 1/33), *S*. *epidermidis* (6%, 2/33), and *S*. *haemolyticus* (15.2%, 5/33) were found to be susceptible to all the antibiotics tested. Reduced susceptibility was observed in several other species. One isolate of *S*. *pasteuri* (3%, 1/33) and four isolates (12.1%, 4/33) of *S*. *warneri* exhibited resistance to fusidic acid with a MIC of 0.5 μg/mL. Additionally, seven isolates of *S*. *pasteuri* (21.1%, 7/33) were tested positive for a cefoxitin screen, indicating methicillin resistance. For *S*. *warneri*, two isolates (6%, 2/33) were resistant to trimethoprim/sulfamethoxazole with an MIC of 80 μg/mL. Regarding the *Pseudomonas spp*. Isolates (*n* = 13), a resistant phenotype was identified in *P*. *medocina*. Two isolates (15.4%, 2/13) showed resistance to the third‐generation cephalosporin ceftazidime (MIC 32 μg/mL), two isolates (15.4%, 2/13) were resistant to imipenem (MIC 32 μg/mL), and three isolates (23.1%, 3/13) exhibited reduced susceptibility to meropenem, with MICs ranging from 4 to 8 μg/mL. In addition, two isolates (15.4%, 2/13) were resistant to piperacillin (MIC 128 μg/mL). In *P*. *stutzeri*, two isolates (15.4%, 2/13) demonstrated resistance to ciprofloxacin (MIC 4 μg/mL), levofloxacin (MIC 8 μg/mL), and piperacillin (MIC 128 μg/mL). An isolate of *E*. *coli* was found to be resistant to several antibiotics, including ampicillin (MIC 32 μg/mL), levofloxacin (MIC 64 μg/mL), and piperacillin/tazobactam (MIC 0.5 μg/mL). Additionally, this isolate exhibited resistance to trimethoprim/sulfamethoxazole (MIC 320 μg/mL). The unique *P*. *agglomerans* was found susceptible to all the antibiotics tested. A full summary of the antimicrobial susceptibility tests is provided in Table S1.

**Figure 6:**
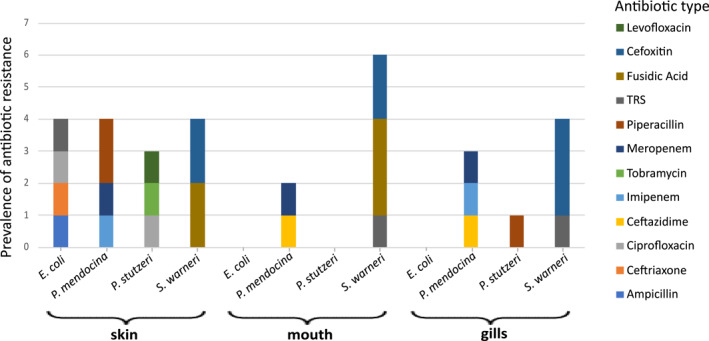
Prevalences of antibiotic resistance among bacterial isolates from different body sites: skin, mouth, gills. TRS = trimethoprim/sulfamethoxazole.

Overall, 31 different reduced susceptibilities were detected from 10 guitarfish, seven females and three males. The skin samples accounted for the highest number of resistant isolates, as well as the greatest variety of antibiotics to which resistance was observed, followed by those from the gills and the mouth (Table S4 and Figure 6).

**Figure 7:**
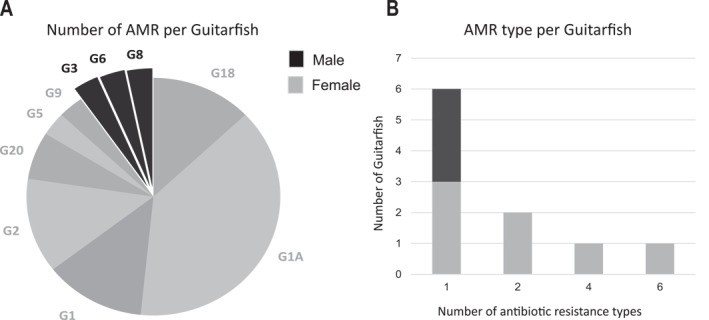
(A) Distribution of antibiotic resistances. Number of distinct AMR detected per individual (*N* = 31) (A), and distribution of individuals (*N* = 10) by the diversity of resistance types detected, categorized by sex.

Although the number of antibacterial resistances differed between sex (Figure 4), no statistically significant differences were found (*p* = 0.650), and activity levels among active individuals also yielded non‐significant results (*U* = 18.0, *p* = 0.084). Nonetheless, a trend toward higher activity in females was observed, suggesting that a larger sample size may be needed to verify this pattern. Notably, the female individual G1A was the only one in which bacteria showed resistance to six different antibiotics. Among males, only three individuals (G3, G6, and G8) carried resistant bacteria. Most individuals showed resistance to only one antibiotic (*n* = 6) (Figure 7).

Among the seven resistance genes tested, only the *bla*_CTX‐M_ gene was detected in nine samples, All the samples were similar and identified as CTX‐M‐185 variant. The positive samples originated from six individual guitarfish (three females and three males), including six skin samples and three gill samples (Table 2).

**Table 2:** Detection of *bla*_CTX‐M_ Gene

Guitarfish ID	Organ	Sex	Gene variant identified
G18	skin	Female	*bla*_CTX‐M‐185_
G18	gills	Female	*bla*_CTX‐M‐185_
G19	skin	Male	*bla*_CTX‐M‐185_
G19	gills	Male	*bla*_CTX‐M‐185_
G2	skin	Female	*bla*_CTX‐M‐185_
G41	skin	Female	*bla*_CTX‐M‐185_
G42	gills	Male	*bla*_CTX‐M‐185_
G42	skin	Male	*bla*_CTX‐M‐185_
G44	skin	Male	*bla*_CTX‐M‐185_

*Note*. CTX‐M positive samples (*n* = 9) from skin (*n* = 6), gills (*n* = 3) and their genetic variant.

**Figure 8:**
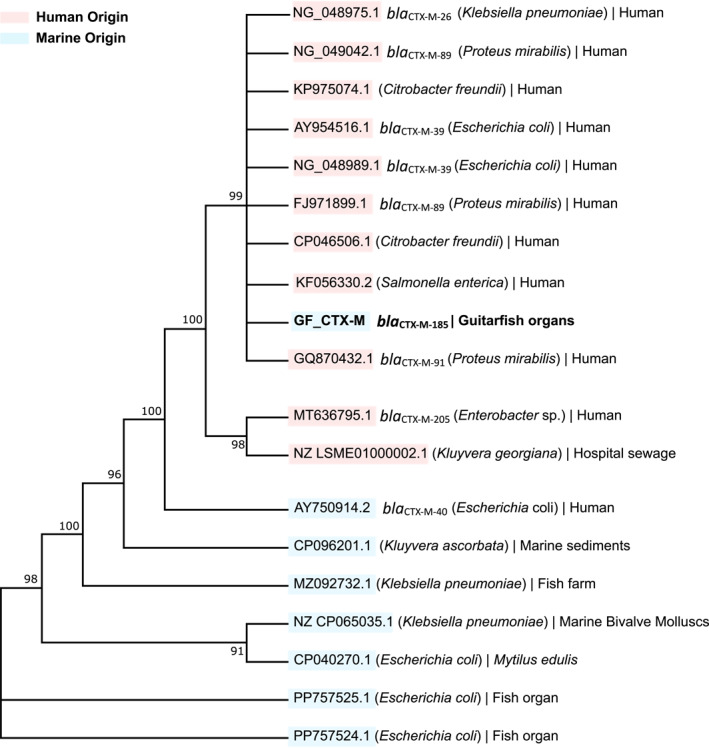
Phylogenetic tree of blaCTX‐M gene sequences, constructed using accession numbers retrieved from NCBI. The bacterial species, the gene variant (if classified) and the source of isolation (e.g., human, marine host etc.) are indicated alongside each accession number. The sequence obtained in this study (GF_CTX‐M) is shown in bold. Sequences derived from human sources are marked in red, and those of marine origin are marked in blue. Numbers on the branches represent phylogenetic distances calculated by the Maximum Likelihood method.

In comparison with closely related sequences obtained from public databases, the sequence was found to be closer to sequences isolated from human‐associated sources than to those from marine environments (Figure 8).

## 4 Discussion

### 4.1 Ecological Context and Relevance in *G*. *cemiculus*

Research on *G*. *cemiculus* in the eastern Mediterranean remains limited, particularly in relation to microbial ecology and environmental health. Studies on this species have focused on physical and ecological parameters such as distribution (Akyol & Capape, 2014; Filiz et al., 2016), age and growth (Başusta et al., 2020), reproductive biology (Bengil et al., 2020; Diatta et al., 2004), diet, feeding habits (Bengil et al., 2020; Correia et al., 2023), and nursery ground (Azrieli et al., 2024).

Our study contributes to the knowledge by evaluating prevalence of AMR analysis into the context of marine conservation and public health. The sampled individuals were neonates, as evidenced by their size (TL), aligning with prior findings that identify the Ma'agan Michael coastline as a potential nursery ground for the species (Azrieli et al., 2024). This life stage is particularly relevant for environmental monitoring, as young individuals are more vulnerable to anthropogenic stressors that negatively impact their habitats, leading to potential declines in fish populations (Toft et al., 2018). Most individuals were sampled from Ma'agan Michael, but additional individuals were also collected from Acer and Evtach, coastal areas subject to different local environmental pressures. Israeli seawater and adjoining coastal waters are mainly affected by biological blooms, chemical contamination, and physical turbidity, all arising predominantly from wastewater discharges and nutrient loading (Nica et al., 2024). Ma'agan Michael coastline is located at the outlet of the Taninim Stream, which is known to be contaminated by wastewater origination primarily from the nearby town of Jisr Az‐Zarqa (Israel Ministry of Environmental Protection, 2026, Taninim Stream). Evtach is a marine reserve situated between the heavily industrialized coastal zones of Ashdod and Ashkelon, making it a key site for assessing the influence of nearby human activity on marine microbial communities (Mafish, 2025). Acer is a coastal city in Israel where municipal sewage influents contain notable levels of nonionic surfactants, indicating a potential risk of contamination in its coastal waters (Zoller & Hushan, 2000), and the outlet of the river Na'aman which is occasionally contaminated as well (Israel Ministry of Environmental Protection, 2023, Na'aman stream). Although each sampling site is characterized by distinct pollution sources, the limited sample size prevents drawing definitive conclusions regarding geographic contrasts or the influence of local pollution on the distribution and diversity of resistant bacteria and genes in marine wildlife.

### 4.2 Anatomical Site‐Specific Exposure and AMR Distribution

We collected samples from three anatomical sites: skin, gills, and mouth representing a different interface with the environment. The skin, constantly exposed to seawater and environmental contaminants, likely serves as a reservoir for antibiotic‐resistant bacteria, as shown in previous studies where the skin mucosal surface retained resistance genes even after antibiotic exposure (Bell et al., 2024; Li et al., 2024). This may explain why skin samples yielded the highest number and diversity of resistant isolates. The gills, involved in gas exchange, are protected by mucosal immunoglobulins and filtration mechanisms that are associated with limit microbial colonization (Xu et al., 2016). The mouth, though internal, is regularly exposed to seawater through feeding and respiration, was showing intermediate levels of resistance compared to the other anatomical sites, also in free‐ranging bottlenose dolphins (*Tursiops truncatus*) (Robles‐Malagamba et al., 2020). These differences highlight the importance of sampling multiple anatomical sites to better understand microbial exposure and AMR distribution in marine species.

### 4.3 Methodological Considerations: Culture‐Based Approaches and MALDI‐TOF MS Limitations

While 16S rRNA gene sequencing and other culture‐independent methods offer a broader view of the taxonomic composition of microbial communities without the need to grow bacteria (Stefani et al., 2015), they do not provide direct information on functional traits such as antibiotic resistance or the presence of specific resistance genes. Isolating bacteria from environmental samples enables not only precise identification, potentially at the whole genome level, but also phenotypic characterization through antibiotic susceptibility testing and genomic analysis of resistance determinants, including plasmids and mobile genetic elements (Kunhikannan et al., 2021). This allows for deeper insights into the functional and ecological potential of bacterial isolates, including their ability to survive in specific environments, interact with hosts, and participate in gene transfer networks, dimensions that cannot be fully assessed through sequencing alone. However, this method is known to be limited in its ability to reflect the full microbial diversity, as only a small fraction of environmental bacteria is capable of growing under standard laboratory conditions. In general, it is estimated that culture‐based methods recover less than 1% of the total microbial community (Sibley et al., 2011). This methodological constraint emphasizes the importance of integrating culture‐independence methods in future studies, as it helps to gain a more comprehensive understanding of microbial diversity and resistance profiles in marine environments (Demko et al., 2021).

We identified approximately half of the bacterial isolates (88 out of 162), which is likely due to the limitations of MALDI‐TOF MS's database when applied to marine and environmental bacteria. MALDI‐TOF MS identification of fish‐associated bacteria is reported in several studies to yield species‐level success rates ranging from roughly 41% to over 95% in select taxa. In many cases, and notably for marine environmental isolates, species‐level identification rates are near 50% (Burbick et al., 2018; Chen et al., 2014; Pérez‐Sancho et al., 2018; Piamsomboon et al., 2020). A key limitation in using MALDI‐TOF MS for environmental and marine microbiology is the lack of comprehensive reference databases. This gap poses a significant challenge as the study of microbial communities in aquatic and marine systems becomes increasingly important within ecological and public health research. Expanding existing databases to better represent environmental taxa is essential to improve the accuracy and reliability of MALDI‐TOF MS in these contexts.

### 4.4 Clinical Relevance, AMR Profiles and One Health Implications

Our study identified several bacterial species with clinical relevance to humans, highlighting the potential health risks associated with AMR in marine environments. The pathogens identified in our study are prevalent in healthcare settings, such as hospitals, significantly contributing to nosocomial infections. These pathogens, including *Staphylococcus spp*., which are typically a part of a natural skin flora in certain cases responsible for a wide range of diseases in immuno‐compromised patients. *S*. *aureus* was also identified, and his related clinical manifestations can vary from less severe skin and soft tissue infections to severe, life‐threatening conditions like bloodstream infections, pneumonia, and endocarditis (Woh & Zhang, 2025). Such diseases are associated with increased morbidity and mortality rates, with some studies indicating that hospital‐acquired infections can lead to a mortality rate increase of up to 15% (Woh & Zhang, 2025). In our study, the *S*. *aureus* isolates were methicillin‐susceptible (MSSA), showing susceptibility to all antibiotics tested. MSSA is responsible for a significant portion of *S*. *aureus* infections in hospital settings. For instance, MSSA is identified in 30%–50% of *S*. *aureus* isolates in hospital settings (Chen et al., 2019; Pardos de la Gandara et al., 2015). The reduced susceptibilities observed in *S*. *pasteuri* and *S*. *warneri*, particularly for fusidic acid and cefoxitin, is concerning as it limits therapeutic options. Approximately 30% of coagulase‐negative staphylococci have been reported to exhibit methicillin resistance, complicating treatment regimens (Esposito & Noviello, 2003). The dominance of this family in marine environments has also been reported in other studies, including in seafood along the northern coast of Greece (Regecová et al., 2014) and in aquaculture facilities across Turkey, where *S*. *aureus* was isolated as well (Çanak & Timur, 2020).

*Pseudomonas spp*., notably *P*. *aeruginosa*, are inherently resistant to many antibiotics. Studies have shown a range of about 10%–21% of *P*. *aeruginosa* clinical isolates that demonstrate carbapenem resistance (Eyo et al., 2015; Farhan et al., 2019; Lin et al., 2016). These phenotypes were particularly challenging because carbapenems are often considered last‐resort treatments for severe infections (Hawkey & Livermore, 2012). The resistance to these drugs limits physicians' ability to effectively treat infections, leading to increased morbidity and mortality rates (Sotello et al., 2018). Furthermore, *P*. *aeruginosa* is known for its ability to resist multiple antibiotics and can transfer resistance genes to other bacteria, compounding the challenges of managing the infections caused by this pathogen the challenges of controlling antibiotic resistance in clinical settings (Silva et al., 2020). The phenotypes were also found in *P*. *medocina* and *P*. *stutzeri*, resistant to ceftazidime, imipenem, and ciprofloxacin suggesting an alarming prevalence of multi‐drug resistant clones. *P*. *mendocina* belongs in the family Pseudomonadaceae and has been isolated from water and soil. Even though it is thought to cause infections quite rarely in humans, it can cause severe infections such as endocarditis, sepsis and central nervous system infections, even in immunocompetent individuals (Ioannou & Vougiouklakis, 2020).

In addition, our study highlighted the presence of a multidrug‐resistant *E*. *coli* isolate, resistant to key antibiotics such as β‐lactams and trimethoprim/sulfamethoxazole. Extended‐spectrum beta‐lactamase (ESBL)‐producing *E*. *coli* poses significant challenges in clinical settings due to its ability to hydrolyze a wide range of beta‐lactam antibiotics, including ampicillin, ceftriaxone and Trimethoprim/Sulfamethoxazole (Silago, 2021). Studies have shown that ESBL‐producing strains can account for over 10% of *E*. *coli* isolates in some hospitals (Denisuik et al., 2019), with carbapenem‐resistant *E*. *coli* strains being identified in 2%–5% of cases (Lodise et al., 2017).

Members of the *Bacillus cereus* group, within the Bacillaceae family, were also among the more abundant isolates recovered. These bacteria are known to cause gastrointestinal illnesses characterized by emetic or diarrheal symptoms and may also cause respiratory and cutaneous infections in immunocompromised individuals (Esmkhani & Shams, 2022; Haque et al., 2021). The presence of Bacillaceae in our samples is consistent with previous environmental studies reporting their detection in marine sediments in China (Liu et al., 2017) and in marine soil samples from the Red Sea coast in Saudi Arabia (Bahamdain et al., 2015).

A study that was conducted in the Levantine basin in the eastern Mediterranean Sea, revealed a high prevalence of antibiotic resistance among Gram‐negative bacteria isolated from sea turtles, with *Vibrio spp*. As a key pathogen. These isolates exhibited significant resistance to several beta‐lactam antibiotics, with a high multidrug resistance rate of up to 75% in some of the isolates (Bachmann et al., 2025). Unlike the turtles sampled in that study, which were mostly diseased, the individuals in our study were juveniles appeared clinically healthy and can also suggest that exposure time to environment plays a role in AMR presence. Detection of AMR in animals which are asymptomatic carriers, highlights their potential role as silent reservoirs and vectors of infection (Endale et al., 2023).

In addition, the detection of blaCTX‐M highlighted the presence of mobile genetic elements. CTX‐M‐185 gene encodes a variant gene of CTX‐M‐type ESBL, which hydrolyze third‐generation cephalosporins such as cefotaxime and ceftriaxone, suggesting a potential reduction in their clinical efficacy. CTX‐M‐185 belongs to the CTX‐M‐25 group, one of the major phylogenetic families within the CTX‐M family (Mendonça et al., 2022). This enzyme was identified in *E*. *coli* isolates from China and has been linked to resistance in community and hospital settings. Its emergence highlights the ongoing diversification of ESBLs under selective pressure from antibiotic use and the importance of surveillance to track novel β‐lactamase variants. These strains lead to limited treatment options and poorer outcomes, with some studies indicating an increase in mortality rates by as much as 50% for infected patients (Allocati et al., 2013; Riley, 2020). *E*. *coli* was found in seawater and fecal samples from otters, seals, and porpoises in the Salish Sea (Grunwald et al., 2022); in sediments along the coast of Helsingborg, Sweden (Erb et al., 2024); and in marine mammals in the Baltic and North Seas (Gross et al., 2022).

Together, these findings emphasize the prevalence of clinically significant pathogens in marine environments and support the growing need to monitor AMR in these systems as part of a broader One Health approach, given the risk of horizontal gene transfer between environmental and human‐associated microbes.

## 5 Conclusions

The findings of this study align with the One Health approach as they demonstrate how AMR, typically considered a clinical or agricultural issue, also exists and spreads within the marine environment, which connects human activity (e.g., wastewater, pollution), animal health (e.g., marine wildlife), and ecosystem stability. The detection of clinically relevant drug‐resistant bacteria in wild marine animals suggests that human‐driven antibiotic use and its persistent presence in the water elevates the risk of marine organisms developing AMR due to ongoing selective pressure and gene dissemination. Environmental contamination directly impacts wildlife, and makes the marine environment an important, and often overlooked, reservoir of AMR. These findings underscore the relevance of marine ecosystems in the broader public health context, as environmental AMR reservoirs may contribute to the reintroduction of resistant bacteria and resistance genes into human‐associated settings.

It is known that aquatic organisms, such as guitarfish, can act as reservoirs and amplifiers of antibiotic resistance genes and resistant bacteria. Their microbiota may facilitate horizontal gene transfer, allowing for the spread of resistance traits within and between species. In this context, *Glaucostegus cemiculus* emerges as a promising sentinel species for AMR surveillance, integrating environmental exposure over space and time and providing early warning signals of anthropogenic AMR pressure in coastal ecosystems.

These findings also highlight the importance of continued monitoring of the marine environment to track AMR dynamics and to inform conservation strategies aimed at safeguarding marine ecosystems. From a One Health perspective, our results support the integration of marine AMR surveillance into national and regional AMR action plans, alongside strengthened wastewater treatment and pollution mitigation strategies to reduce antibiotic and resistant bacteria release into coastal waters. Such measures are essential to protect marine biodiversity, limit environmental amplification of AMR, and mitigate downstream risks to human and animal health.

## Conflict of Interest

The authors declare no conflicts of interest relevant to this study.

## Supporting information

Table S1

Table S2

Table S3

Table S4

## Data Availability

All novel blaCTX‐M gene sequences generated in this study are being deposited in NCBI GenBank (accession numbers PX856897–PX856905). All other data supporting the findings of this study, including tables, four supplementary tables, and additional figures, are provided within the article and its Supporting Information files. In addition, the underlying data set supporting these analyses is publicly available in the Zenodo repository (Lellouche et al., 2026). The data set DOI is: [https://doi.org/10.5281/zenodo.19029073] [Dataset].
